# Precipitous Increase of Bacterial CRISPR-Cas Abundance at Around 45°C

**DOI:** 10.3389/fmicb.2022.773114

**Published:** 2022-03-01

**Authors:** Xin-Ran Lan, Zhi-Ling Liu, Deng-Ke Niu

**Affiliations:** MOE Key Laboratory for Biodiversity Science and Ecological Engineering and Beijing Key Laboratory of Gene Resource and Molecular Development, College of Life Sciences, Beijing Normal University, Beijing, China

**Keywords:** CRISPR-Cas, optimal growth temperature, bacteria, protistan grazing, viral lysis, mortality

## Abstract

Although performing adaptive immunity, CRISPR-Cas systems are present in only 40% of bacterial genomes. We observed an abrupt increase of bacterial CRISPR-Cas abundance at around 45°C. Phylogenetic comparative analyses confirmed that the abundance correlates with growth temperature only at the temperature range around 45°C. From the literature, we noticed that the diversities of cellular predators (like protozoa, nematodes, and myxobacteria) have a steep decline at this temperature range. The grazing risk faced by bacteria reduces substantially at around 45°C and almost disappears above 60°C. We propose that viral lysis would become the dominating factor of bacterial mortality, and antivirus immunity has a higher priority at higher temperatures. In temperature ranges where the abundance of cellular predators does not change with temperature, the growth temperatures of bacteria would not significantly affect their CRISPR-Cas contents. The hypothesis predicts that bacteria should also be rich in CRISPR-Cas systems if they live in other extreme conditions inaccessible to grazing predators.

## Introduction

CRISPR-Cas systems provide adaptive immunity against mobile genetic elements for bacteria and archaea. Like the adaptive immune system of jawed vertebrates, the CRISPR-Cas systems can remember previously encountered pathogens, initiate a rapid response to a second invasion, and eliminate the recurrent invader. However, unlike the ubiquitous presence of adaptive immunity in jawed vertebrates (Müller et al., 2018), the CRISPR-Cas systems are only present in about 40% of bacteria (Makarova et al., 2020). The patchy distribution of the CRISPR-Cas systems among bacteria is a recognized mystery (Ledford, 2017; Koonin, 2018). Given the constant horizontal transfers, their absence in more than half of bacterial genomes is unlikely to happen just by chance (Bernheim, 2017). Instead, it should be attributed to a tradeoff between the costs and benefits of the CRISPR-Cas systems. First, the acquirement and maintenance of CRISPR-Cas systems would sequestrate limiting resources such as the building blocks, the energy, and the transcription and translation machines (Lynch and Marinov, 2015; Vale et al., 2015; Frumkin et al., 2017). Second, the autoimmune response and cell death induced by self- and prophage-targeting spacers might be a selective force for the loss of CRISPR-Cas systems (Rollie et al., 2020; Wimmer and Beisel, 2020). In addition, for the viruses with high densities, high mutation rates, high genetic diversities, or carrying anti-CRISPR proteins, the efficiency of CRISPR-Cas systems is limited (Weinberger et al., 2012; Iranzo et al., 2013; Westra et al., 2015; Trasanidou et al., 2019). CRISPR-Cas systems are not favored in conditions with high antibiotic pressures because they inhibit horizontal gene transfer, an efficient way for bacteria to acquire antibiotic resistance (Palmer and Gilmore, 2010).

Just after discovering the CRISPR-Cas structures, it was noticed that they are more prevalent in the thermophilic archaea and the hyperthermophilic bacteria (Jansen et al., 2002; Makarova et al., 2002). Later large-scale analyses confirmed the prevalence of CRISPR-Cas systems in thermophiles and hyperthermophiles and showed a positive correlation between CRISPR abundance and growth temperatures (Anderson et al., 2011; Makarova et al., 2011; Weinberger et al., 2012; Gophna et al., 2015). Recently, Weissman et al. (2019) tried a phylogenetically corrected machine learning approach using models like logistic regression, sparse partial least squares discriminant analysis, and random forest with the data split into different blocked folds by their pairwise distance on the phylogeny. Their results indicate that temperature and oxygen levels might be the most influencing ecological factors determining the distribution of the CRISPR-Cas systems. In this paper, we describe an abrupt increase of bacterial CRISPR-Cas systems at around 45°C and put forward a new hypothesis on the thermal distribution of the CRISPR-Cas systems.

## Materials and Methods

The phylogenetic relationships among the analyzed species were retrieved from Genome Taxonomy Database (GTDB) (Parks et al., 2021). The GTDB group constructed the phylogenetic tree of bacteria and archaea using constantly updated whole-genome sequences. They selected a representative genome for each species. The sample size of their representative genomes is similar to the NCBI genome database.^1^ For the accuracy in estimating the CRISPR-Cas abundance, we only retrieved the representative genomes assembled at the levels of “complete genome” or “chromosome” from the GTDB database (accessed: Dec. 24, 2021), 4908 bacterial and 292 archaeal genomes. Then, we downloaded the genome sequences from ftp://ftp.ncbi.nlm.nih.gov/genomes/ and annotated the CRISPR-Cas systems using CRISPRCasFinder v1.3 (Couvin et al., 2018). According to Couvin et al. (2018), the annotated CRISPR arrays were classified into four categories, 1 to 4, according to their evidence levels. The CRISPR arrays with evidence levels 3 and 4 are highly likely candidates, and those with evidence levels 1 and 2 are potentially invalid. Therefore, only the CRISPR arrays with evidence levels 3 and 4 were counted in calculating CRISPR array abundance. We counted the putative CRISPR arrays with evidence levels 1 and 2 as zero. The abundance of an object in a genome was defined as the simple count of the object (CRISPR arrays, CRISPR spacers, cas genes, or cas gene clusters) in the genome.

Based on these 5200 representative genomes, we constructed three datasets.

The first is the smallest but most accurate, including 1351 species (1168 bacteria and 183 archaea, Supplementary Table 1). This dataset was constructed by retrieving the direct links of Genbank IDs and optimal growth temperature of the same strain from the database TEMPURA (Sato et al., 2020) and the reference (Lyubetsky et al., 2020), and then manually matching the strain names of the other records in TEMPURA with the complete genomes deposited in Genbank. In this dataset, the Topt and the genome of each species must come from the same strain. Potential errors resulting from polymorphism of Topt or CRISPR abundance within each species have been eliminated.

The second dataset contains 3154 genomes (2944 bacteria and 210 archaea, Supplementary Table 2). In constructing this dataset, polymorphisms among the strains of a single species were neglected. The strain name of each species was overlooked. The Topts were retrieved from a series of sources. For the conflicting records of Topt between different resources, the preferential rank was from the Topts with GenBank IDs in TEMPURA (Sato et al., 2020) and the reference (Lyubetsky et al., 2020), the Topts in reference (Madin et al., 2020), the Topts not associated with GenBank IDs in TEMPURA, the growth temperatures in reference (Madin et al., 2020), the growth temperatures in the reference (Engqvist, 2018), to the growth temperatures in BacDive (Reimer et al., 2018).

The third dataset contains all the 5200 representative genomes retrieved from GTDB (Supplementary Table 3). The Topt values of these species were predicted using a machine-learning method (Tome, version 1.0.0) reported in reference (Li et al., 2019).

All the temperature values analyzed in this study were first rounded into integers.

These three datasets gave similar column charts for the relationships between Topt and CRISPR-Cas abundance. We presented the charts from the 3154 genomes in the main text, and all further analyses were based on this dataset. The charts obtained from the other two datasets were deposited in Supplementary Tables 4, 5.

The presence or absence of each prokaryote in particular environmental conditions was obtained from the isolation source data of the BacDive database (Reimer et al., 2018). The restriction-modification (RM) enzyme genes in each genome were obtained from the PADS Arsenal database (Zhang et al., 2020). All these data were deposited in Supplementary Table 2.

The phylogenetic signals (λ) of CRISPR array abundance, CRISPR spacer abundance, *cas* gene abundance, *cas* gene cluster abundance, and growth temperatures were estimated using the phylosig function of the R (Version 4.0.3) package phytools (Version 0.7-70) (Revell, 2012). The phylogenetic generalized least squares (PGLS) regression was performed using the R (Version 4.0.3) package phylolm (version 2.6.2) (Ho and Ane, 2014). Pagel’s λ model has been applied in the analyses.

The non-linearity of the relationships was estimated using the generalized additive model (GAM) that was integrated into the R package mgcv (version 1.8-33) (Wood, 2017). The derivative of the GAM curve was calculated using the R package gratia (Version 0.6.0) (Simpson, 2022).

## Results

### Bacterial CRISPR-Cas Abundance Increases Precipitously at Around 45°C

**FIGURE 1:**
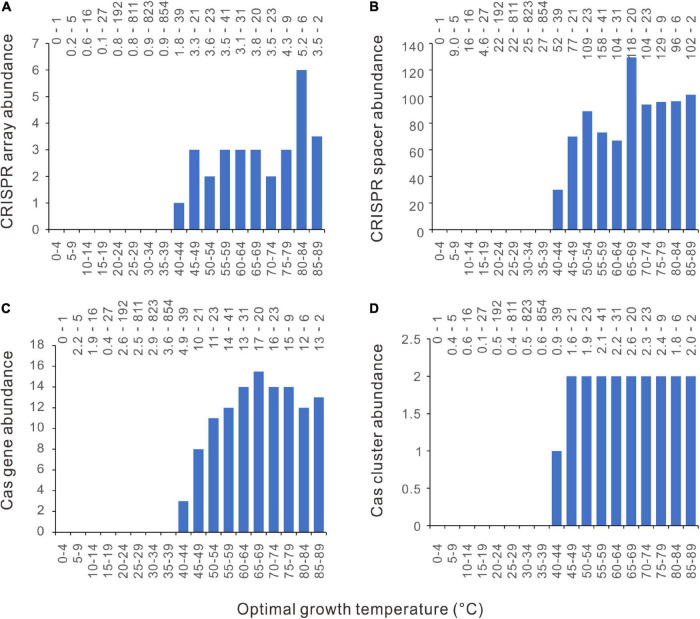
Distinctive relationships of bacterial CRISPR-Cas abundance with different optimal growth temperature (Topt) ranges. The abundances of CRISPR arrays **(A)**, spacers **(B)**, *cas* genes **(C)**, and *cas* gene clusters **(D)** increase substantially with Topt at around 40–45°C. Because median values are less sensitive to extreme outliers than the mean values, we created the column charts using the median value of each Topt range. The mean value and the number of genomes of each bin (separated by a hyphen) are shown at the top of each chart.

We examined the thermal distribution of bacterial CRISPR-Cas abundance by calculating the median value of the optimal growth temperatures (Topt) in each five-degree temperature range bin. From 4 to 85°C, the temperature range of the 2944 bacteria was divided into 18 bins. By plotting the CRISPR-Cas abundance against the Topt in column charts, we see a novel pattern on the thermal distribution of CRISPRs in bacteria (Figure 1A). An abrupt increase of bacterial CRISPR array abundance happens at 40−45°C. The CRISPR array abundance fluctuates above 45°C, but the amplitudes of the fluctuations are much weaker. In all the bins below 40°C, the median values of CRISPR array abundance are consistently zero. When measured by the mean values, the CRISPR array abundances of all the bins below 40°C are <1, whereas those above 45°C are >3. At around 40−49°C, precipitous increases could also be observed in the abundance of bacterial CRISPR spacers, *cas* genes, and *cas* gene clusters (Figures 1B–D). In summary, the CRISPR-Cas-poor and rich bacteria are concentrated in low and high temperatures, respectively, and the transitions from CRISPR-Cas poor to CRISPR-Cas rich occur at a narrow range, like 40−49°C.

The separate distribution of CRISPR-Cas poor and CRISPR-Cas rich bacteria along the temperature axis might be attributed to a strong effect of temperature on CRISPR-Cas system evolution. It is also possible that the results happen just by chance. To test this possibility, we performed a permutation test by randomly shuffling the 2944 bacteria along the axis of Topt 1000 times. The null hypothesis is that the bacteria were randomly distributed within the studied temperature range. To compare the observed results with the null hypothesis, we first quantified the difference of CRISPR-Cas abundance (*D*) between low and high temperatures:

$$D_{i}={\text{Average}}_{(i,\text{max\{Topt\})}}\left(\text{CRISPR}-\text{Cas}\right)/{\text{Average}}_{(\text{min\{Topt\},}i\text{-1)}}\left(\text{CRISPR}-\text{Cas}\right)$$

**FIGURE 2:**
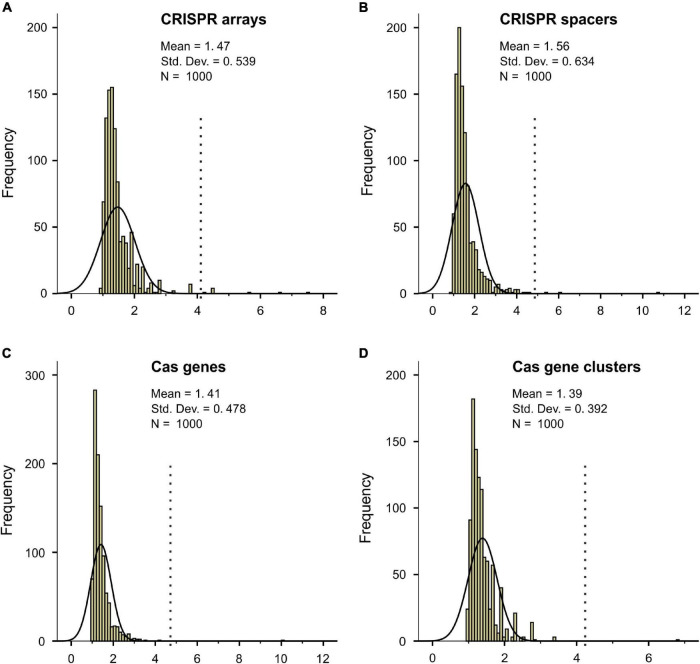
Permutation tests for the separate distribution of CRISPR-Cas poor and CRISPR-Cas rich bacteria along the temperature axis. The null hypothesis is that the bacteria were randomly distributed within the studied temperature range. The ratio of average CRISPR-Cas abundance at equal and above a temperature divided by that below the temperature was defined as *D*_i_. The maximum values of *D*_i_ for the observed results (indicated by the dotted lines) and the 1000 rounds of random shuffling of CRISPR arrays, spacers, *cas* genes, and *cas* gene clusters were shown in **(A–D)**.

We used the average values rather than the median values because more than 50% of bacterial genomes lack CRISPR-Cas systems. By plotting the *D*_i_ against Topt, we could see that it varies significantly at the two ends of the Topt axis (Supplementary Figure 1A for the observed CRISPR arrays and Supplementary Figure 1B for a randomly shuffled sample), probably because of the small sample size at very low and very high temperatures. To minimize the random noise resulting from the average values of small-size samples, we retained only the *D*_i_ from 20 to 64°C. Within this range, the maximum value of the difference, *D*_max_, for the observed CRISPR arrays is 4.13. Among the 1000 rounds of random shufflings, the more extreme maximum values of *D*_i_ (> 4.13) were obtained in only five rounds (Figure 2A). The permutation test showed that the separate distribution of CRISPR-Cas poor bacteria at low temperatures and CRISPR-Cas rich bacteria at high temperatures is statistically significant (*p* = 0.005). By the same method, we confirmed the statistical significance for the CRISPR spacers, *cas* genes, and *cas* gene clusters (*p* = 0.003, 0.002, and 0.001, respectively) (Figures 2B–D).

**FIGURE 3:**
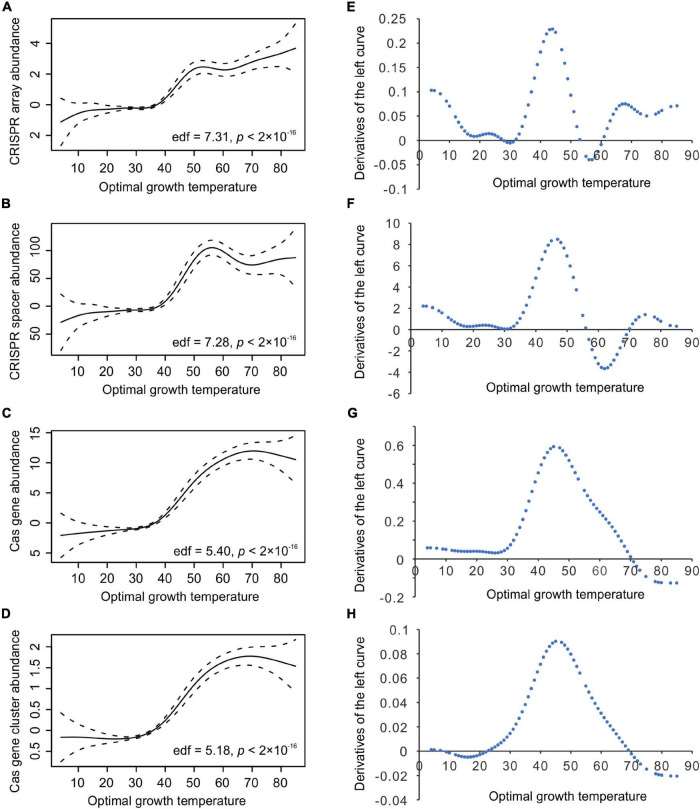
The relationship between bacterial optimal growth temperature (Topt) and the CRISPR-Cas abundance revealed by the generalized additive model (GAM). High non-linearity was detected in the relationship of Topt with CRISPR arrays **(A)**, CRISPR spacers **(B)**, *cas* genes **(C)**, and *cas* gene clusters **(D)**. The derivatives of the GAM curves in **(A–D)** revealed the most abrupt transitions at 44°C **(E)**, 47°C **(F)**, 45°C **(G)**, and 45°C **(H)**, respectively. The effective degrees of freedom (edf) proxy for non-linearity in the relationships.

The column charts (Figure 1) clearly show that the relationship between CRISPR-Cas abundance and Topt is not linear. To capture the critical aspect of the relationship, we fitted the data with the smooth functions of the GAM. As shown in Figures 3A–D, the model could capture the sharp increases of bacterial CRISPR-Cas abundance from about 35 to 50°C. We calculated the derivatives of the GAM curves and presented the results in Figures 3E–H. The temperatures with the highest slope values appeared at 44, 47, 45, and 45°C for the abundances of CRISPR arrays, spacers, *cas* genes, and *cas* gene clusters, respectively. In summary, the sharp increases of bacterial CRISPR-Cas abundances are at around 45°C.

### Phylogenetic Analysis Confirmed the Positive Correlations Between Bacterial CRISPR-Cas and Topt at Around 45°C

Because of the existence of shared ancestors, the data across related species are often not statistically independent and violate one of the most basic assumptions of most standard statistical procedures (Felsenstein, 1985; Symonds and Blomberg, 2014). To evaluate the effects of shared ancestors, we measured the phylogenetic signals (Pagel’s λ) of bacterial CRISPR-Cas abundances and Topt. The λ value ranges from zero to one, where zero indicates statistical independence of the data, whereas one indicates that the data are strongly affected by the shared ancestors. The 2944 bacterial dataset exhibits strong phylogenetic signals (λ = 0.846, 0.922, 0.884, 0.872, and 0.975 for the abundance of CRISPR arrays, spacers, *cas* genes, *cas* gene clusters and Topt, respectively; and the significance value *p* < 10^–275^ for all the five cases).

A series of phylogenetic comparative methods have been developed to control the effects of the shared ancestors (Garamszegi, 2014). We used the PGLS regression to measure the relationships between CRISPR-Cas abundance and Topt. A significant positive slope corresponds to a significant positive correlation, and a negative slope indicates the reverse. Figures 1, 2 showed that the relationships between CRISPR-Cas abundance and Topt are not linear. However, when the data were divided into segments, we could use linear models to estimate the relationships within each segment.

**FIGURE 4:**
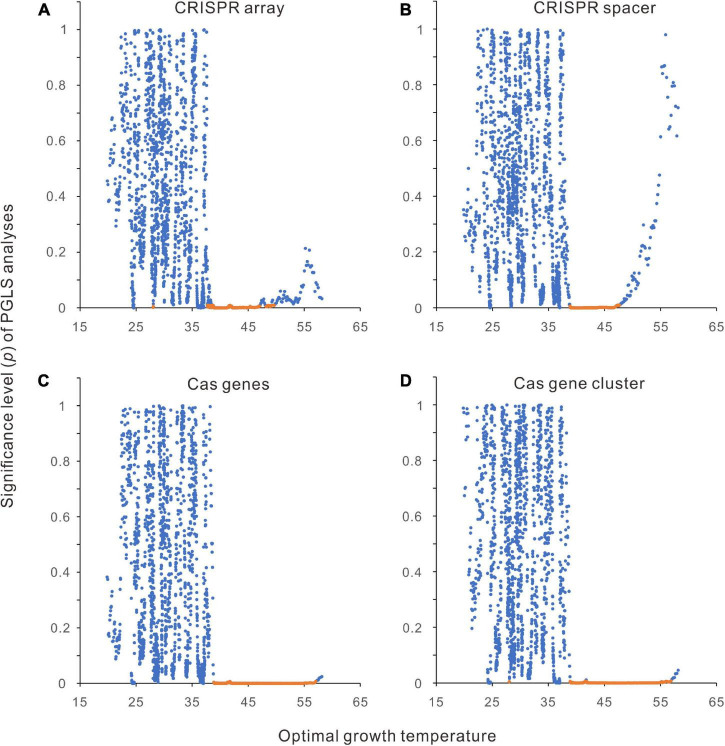
Segmental phylogenetic generalized least squares (PGLS) regression analysis of the relationships between CRISPR-Cas abundances and optimal growth temperatures (Topt). **(A)** CRISPR arrays. **(B)** CRISPR spacers. **(C)** *cas* genes. **(D)** *cas* gene clusters. The 2944 bacteria were aligned along the Topt axis. Two hundred neighboring samples were taken in each round of PGLS analysis. The average Topt of each 200 samples was presented in the scatter charts. The regression results with positive slopes and < 0.01 significance values are orange.

By aligning the 2944 bacteria along the Topt axis, we performed a PGLS analysis for every 200 neighboring samples, except that all the 200 samples had the same Topt. In total, 1840 rounds of PGLS analyses have been performed for each abundance parameter. In such a large-scale analysis, tens of significant correlations (including negative and positive correlations) likely happen by chance if the statistical significance is defined by *p* < 0.05. As shown in Figures 4A–D, all the correlations around 45°C are significant, whereas only a tiny percentage of correlations are significant in low and high-temperature ranges. When the statistical significance was defined more stringently using *p* < 0.01, positive correlations were observed only around 45°C except for one or two cases in CRISPR arrays and cas gene clusters (Figures 4A,D).

Furthermore, we classified the 2944 bacteria into three categories according to their Topts, low temperatures (4 ≤ Topt ≤ 34°C, *n* = 1875), moderate temperatures (35 ≤ Topt ≤ 49°C, *n* = 914), and high temperatures (50 ≤ Topt ≤ 85°C, *n* = 155), significant correlations were only observed in the moderate-temperature bacteria (Table 1). PGLS regression also showed positive correlations when all the 2944 bacteria were analyzed together (Table 1).

**TABLE 1:** Correlations between bacterial CRISPR-Cas abundance and optimal growth temperature.

		CRISPR arrays	CRISPR spacers	*Cas* genes	*Cas* gene clusters
**Bacteria**					
4 − 34°C (*n* = 1875)	Slope	0.012	–0.021	0.021	0.002
	*p*	0.266	0.947	0.499	0.716
35 − 49°C (*n* = 914)	Slope	0.128	2.020	0.277	0.042
	*p*	**7 × 10^–6^**	**0.026**	**0.002**	**0.005**
50 − 85°C (*n* = 155)	Slope	0.026	–0.665	0.046	0.003
	*p*	0.352	0.616	0.645	0.842
4 − 85°C (*n* = 2944)	Slope	0.037	0.764	0.100	0.017
	*p*	**6 × 10^–11^**	**3 × 10^–5^**	**9 × 10^–9^**	**10^–95^**
**Archaea**					
18 − 39°C (*n* = 67)	Slope	0.010	−0.385	0.222	0.036
	*P*	0.813	0.814	0.125	0.075
40 − 80°C (*n* = 75)	Slope	0.086	0.230	0.054	0.006
	*P*	**0.027**	0.851	0.501	0.622
81 − 106°C (*n* = 68)	Slope	0.124	2.119	0.333	0.064
	*P*	0.101	0.207	**0.050**	**0.016**
18 − 106°C (*n* = 210)	Slope	0.087	0.819	0.096	0.017
	*P*	**2 × 10^–6^**	0.087	**0.021**	**0.007**

*P values less than or equal to 0.050 are shown in bold.*

### Precipitous Increase of CRISPR-Cas Abundance at Around 45°C Observed in Diverse Environments

**FIGURE 5:**
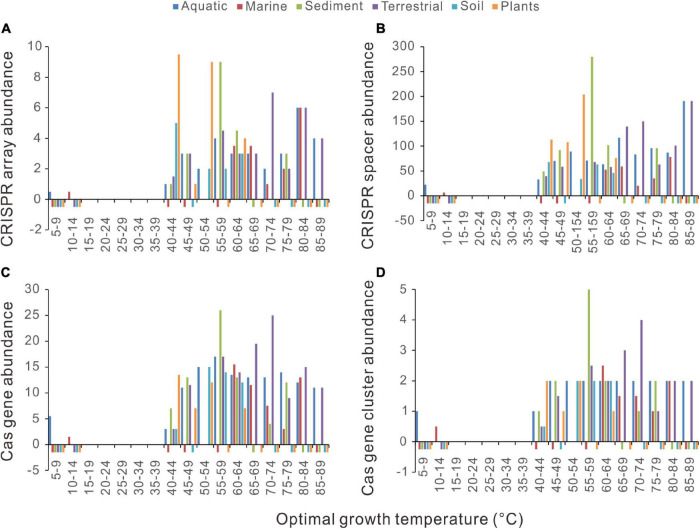
Relationships between CRISPR-Cas abundances and optimal growth temperatures (Topt) in diverse environments. **(A)** CRISPR arrays. **(B)** CRISPR spacers. **(C)** *cas* genes. **(D)** *cas* gene clusters. Because median values are less sensitive to extreme outliers than the mean values, we created the column charts using the median value of each Topt range. The Topt ranges without any samples were marked out using short negative bars.

We retrieved the isolation source category data from the BacDive database (Reimer et al., 2018) and checked the 2944 bacteria. The isolation sources differ significantly in the number of bacteria species and the temperature range. We only selected the isolation source categories containing >150 bacterial species to reduce sample bias. Because we are interested in the sharp increase of CRISPR-Cas abundance at around 45°C, isolation sources were also filtrated using their temperature ranges, with their upper limits not lower than 60°C and their lower limits not higher than 30°C. Six isolation sources were retained using these criteria, including aquatic, marine, sediment, terrestrial, soil, and plants. Although the temperature ranges of some sources are much narrower than that of the 2944 bacteria dataset, sharp increases of CRISPR-Cas abundance at around 45°C could be observed in all the surveyed environmental conditions (Figures 5A–D).

### The Abundance of Bacterial Type I CRISPR-Cas System Jumps Up at Around 45°C

**FIGURE 6:**
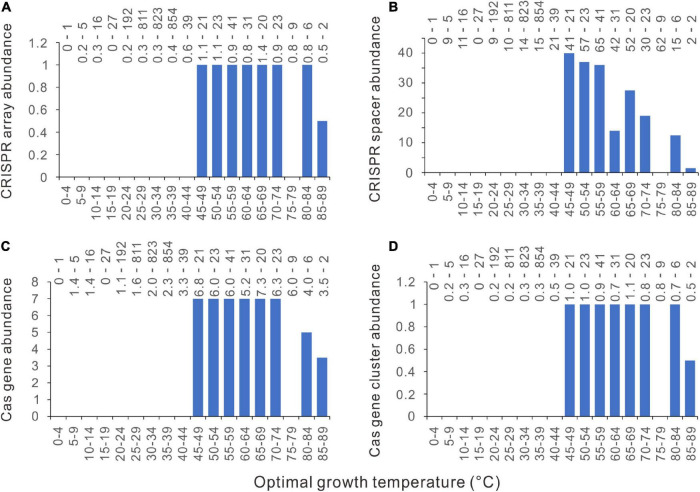
Relationships between Type I CRISPR-Cas abundances and optimal growth temperatures (Topt). **(A)** CRISPR arrays. **(B)** CRISPR spacers. **(C)** *cas* genes. **(D)** *cas* gene clusters. Because median values are less sensitive to extreme outliers than the mean values, we created the column charts using the median value of each Topt range. The mean value and the number of genomes of each bin (separated by a hyphen) are shown at the top of each chart.

Currently, CRISPR-Cas systems are classified into six types (Type I − VI) according to their *cas* gene content and Cas protein sequence conservation (Makarova et al., 2020). The CRISPR-Cas systems that could not be confidently classified into these six types have been labeled as “unknown” by the program CRISPRCasFinder v1.3 (Couvin et al., 2018). The type I CRISPR-Cas system is the most frequent in bacteria and archaea (Bernheim et al., 2019; Makarova et al., 2020). We examined the thermal distribution of these seven groups (Type I − VI and unknown) by plotting the median values in each five-degree temperature range against the Topts. Unexpectedly, among type II, IV, V, VI, and the unknown group, all the bins have consistently median values of zero for CRISPR arrays, spacers, cas genes, and cas gene clusters. These CRISPR-Cas systems are absent from more than half of the analyzed genomes. The type III CRISPR-Cas system has only one bin with median values > 0, 85 − 89°C. The type I CRISPR-Cas system exhibits a thermal distribution pattern similar to but more distinctive than when all the CRISPR-Cas systems were counted together. There are abrupt jumps at 45°C (Figures 6A–D). For instance, the *cas* gene abundance jumps directly from zero to seven at 45°C, without a transitional column at 40 − 44°C.

### Archaeal CRISPR-Cas Abundance Has a Less Distinctive Pattern

**FIGURE 7:**
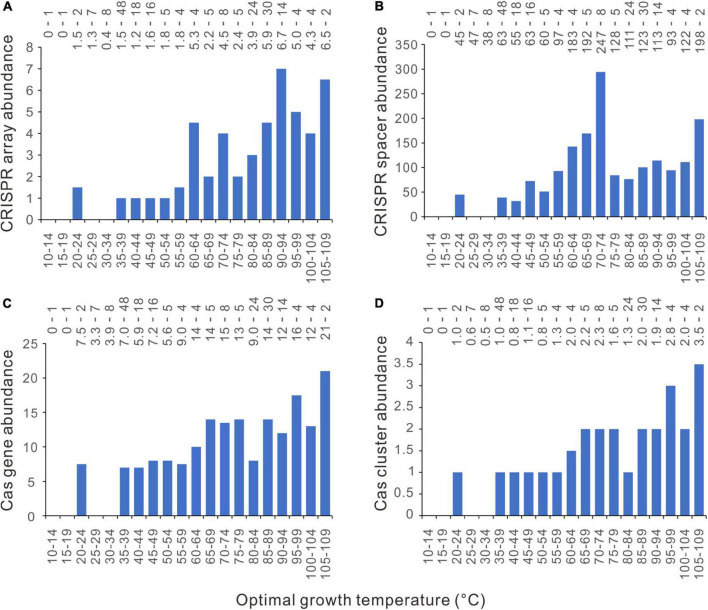
Relationships between archaeal CRISPR-Cas abundances and optimal growth temperatures (Topt). **(A)** CRISPR arrays. **(B)** CRISPR spacers. **(C)** *cas* genes. **(D)** *cas* gene clusters. Because median values are less sensitive to extreme outliers than the mean values, we created the column charts using the median value of each Topt range. The mean value and the number of genomes of each bin (separated by a hyphen) are shown at the top of each chart.

We also examined the relationship between topt and CRISPR-Cas abundances in archaea. Generally, the CRISPR-Cas abundances increase with growth temperature (Figures 7A–D and Supplementary Figure 2). For the CRISPR array, *cas* gene, and *cas* gene cluster, there are no abrupt increases at around 45°C or different temperatures. The CRISPR spacer abundance increases steadily from 40 to 74°C (Figure 7B and Supplementary Figure 2A). The GAM gave almost linear regressions for the abundances of CRISPR arrays, *cas* genes, and *cas* gene clusters (Supplementary Figure 2). These observations were based on small sample sizes of the bins and were sensitive to the presence of a few outliers.

The 210 archaeal dataset also exhibits significant phylogenetic signals (λ = 0.802, 0.591, 0.537, 0.631, and 0.976 for the abundance of CRISPR arrays, spacers, *cas* genes, *cas* gene clusters, and Topt, respectively; and the significance value *p* < 10^–10^ for all the five cases). Overlapping segmental PGLS regression analyses found significant positive correlations of Topt with the CRISPR spacer abundance at around 45°C, but not the abundances of CRISPR arrays, *cas* genes, or *cas* gene clusters (Supplementary Figure 3). Because the 210 archaeal species are enriched in thermophiles and hyperthermophiles, dividing them into three temperature range categories using the above thresholds leads to a too-small sample for the low-temperature category (*n* = 19). Therefore, we divided the 210 archaea into three nearly equal-sized groups according to their Topts (Table 1). PGLS regression showed a significant correlation between Topt and CRISPR array abundance in the moderate-temperature group (*n* = 75, 40 ≤ Topt ≤ 80°C, slope = 0.086, *p* = 0.027), but not in the lower (*p* = 0.813) or the higher one (*p* = 0.101). The *cas* gene abundance and *cas* gene cluster abundance are positively correlated with Topt only in the high-temperature group (Table 1). The CRISPR spacer abundance is not correlated with Topt in all three groups. Although the CRISPR spacer abundance seems to increase steadily from 40 to 74°C, no significant positive correlation was found (slope = 2.07, *p* = 0.165). A significant positive correlation appeared when the CRISPR spacer abundance was sampled from 10 to 74°C (slope = 1.62, *p* = 0.003). When all the 210 archaeal genomes were analyzed together, globally positive correlations were observed between Topt and the abundances of CRISPR arrays, *cas* genes, and *cas* gene clusters (*p* < 0.05 for all three cases). In contrast, the CRISPR spacer abundance and Topt were correlated at marginal level (0.05 < *p* < 0.1, Table 1).

In addition, we examined whether bacteria and archaea living at the same temperature are different in their CRISPR-Cas abundance. We selected the temperatures that are Topts of both bacterial and archaeal species. The CRISPR-Cas abundance of bacteria/archaea with the same Topt were averaged. Pairwise comparison of the obtained 41 archaeal-bacterial pairs found marginally significant differences in CRISPR array and *cas* gene cluster abundances, but not in the abundances of spacers and *cas* genes (Wilcoxon signed ranks test, *p* = 0.049, 0.047, 0.572, and 0.313, respectively). Bacteria have a little more CRISPR-Cas systems than the archaea living at the same temperature.

### No Precipitous Increase of Restriction-Modification Gene Abundance at Around 45°C

**FIGURE 8:**
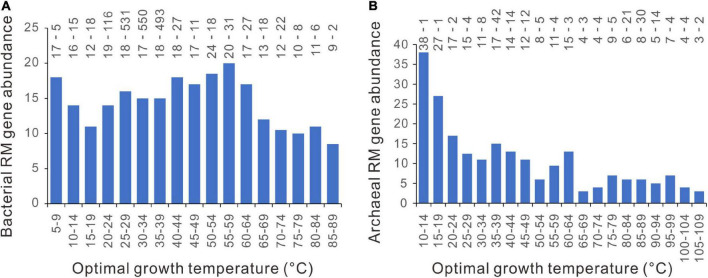
Relationships between restriction-modification (RM) gene abundance and optimal growth temperature. **(A)** bacteria. **(B)** archaea. Because median values are less sensitive to extreme outliers than the mean values, we created the column charts using the median value of each Topt range. The mean value and the number of genomes of each bin (separated by a hyphen) are shown at the top of each chart.

In contrast to the patchy distribution of CRISPR-Cas systems (Makarova et al., 2020), the RM systems are present in about 96% of bacterial genomes and 97% of archaeal genomes (Roberts et al., 2015). We counted the number of RM enzyme genes in each genome and plotted them against the Topt. As shown in Figure 8, there are no abrupt increases at around 45°C. PGLS regression analysis showed that the RM gene abundance significantly declines with Topt, statistically significant in archaea (*n* = 183, slope = − 0.162, *p* = 10^–6^) and marginally significant in bacteria (*n* = 1898, slope = − 0.061, *p* = 0.079).

## Discussion

Previous studies have found that prokaryotes living in high temperatures are more likely to have CRISPR-Cas systems (Jansen et al., 2002; Makarova et al., 2002, 2011; Anderson et al., 2011; Weinberger et al., 2012; Gophna et al., 2015; Weissman et al., 2019). This study presented a more detailed description of the relationship between CRISPR-Cas and Topt. At low (4 − 34°C) and high temperatures (50 − 85°C), the abundances of bacterial CRISPR-Cas are not significantly correlated with Topt. However, at around 45°C, bacterial CRISPR-Cas abundance increases sharply with Topt. Most significantly, the bacterial type I CRISPR-Cas abundance exhibits an abrupt jump at 45°C. As we see, the evolutionary and mechanical links between growth temperature and CRISPR-Cas abundance previously proposed (Weinberger et al., 2012; Iranzo et al., 2013; Høyland-Kroghsbo et al., 2018) could not explain the abrupt transition at around 45°C. Temperature influences many aspects of cellular processes and the physical features of the environment (Clarke, 2014). No matter linear or non-linear, the biological effects of temperature increase gradually.

**FIGURE 9:**
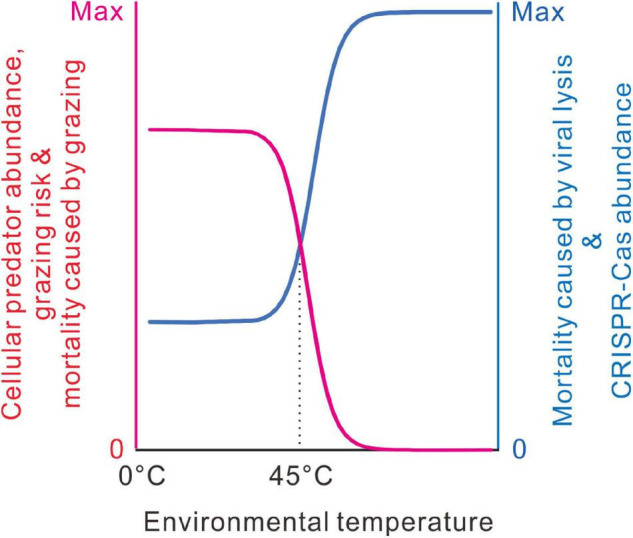
A model for the thermal distribution of bacterial CRISPR-Cas systems. At low temperatures, the evolution of CRISPR-Cas systems is limited by the grazing pressure of cellular predators. At around 45°C, cellular predator abundance decreases abruptly with environmental temperature, so viral lysis takes more in bacterial mortality, and the requirement of the immune function increases substantially. When cellular predators almost disappear at high temperatures, mortality results primarily from viral lysis; most bacteria evolve the highest antivirus capacity.

From the literature, we noticed that the thermal distribution of eukaryotes has an abrupt decline at around 45°C. Very few eukaryotes can live above 60°C (Tansey and Brock, 1972; Brock, 1985, 2001; Clarke, 2014). Bacterial communities are often heavily consumed by eukaryotic predators (Jousset, 2012). In freshwater and marine environments, viral lysis and predation by ciliated and flagellated protists contributed to most bacterial mortality (Pernthaler, 2005; Takasu et al., 2014). In addition, some bacteria could also kill other bacteria and consume the released nutrients. Except for a few exceptions, most predatory bacteria could not grow at temperatures above 45°C (Reichenbach, 1999; Williams and Chen, 2020). Here, we propose that the accessibility of cellular predators along the temperature axis might indirectly govern the thermal distribution of the CRISPR-Cas systems (Figure 9). Cellular predators are the bacterivorous eukaryotes and predatory bacteria, such as protozoa, nematodes, myxobacteria, and *Bdellovibrio*.

In birds, it has been shown that predation risk could significantly reduce the allocation of the limiting resources to immune function (Møller and Erritzøe, 2000). The birds captured by cats consistently had smaller spleens than those killed by non-predatory reasons like collisions with windows or cars. When the hosts are exposed to lethal predators, predator-mediated mortality becomes dominant, and the pathogen-mediated mortality decreases relatively. Consequently, the priority to invest in immune function is reduced. Physiological and evolutionary reducing the allocation of the limiting resources to immune function would be favored.

We proposed that the same case might happen in bacteria. For picophytoplankton, grazer-mediated mortality and viral-mediated mortality are inversely correlated (Pasulka et al., 2015; Staniewski and Short, 2018). Indirect interactions among grazers and viruses are destined to occur, provided that bacterial cells have tradeoffs in grazing and virus resistance. When bacterial mortality mostly comes from predator grazing, the benefits of adaptive immunity might be dwarfed by the costs in the maintenance and expression of the CRISPR-Cas systems, like allocating the limiting resources, targeting host or prophage genome, and inhibiting horizontal gene transfer. By contrast, when bacterial mortality mostly comes from viral lysis, some costs of CRISPR-Cas systems would be tolerable because the benefits of adaptive immunity outweigh the costs. At around 45°C, with the increase of environmental temperature, cellular predator abundance decreases sharply (Brock, 1985; Clarke, 2014); thus, the grazing risk of bacteria should be abruptly relieved. Bacteria growing at higher temperatures should die mainly from viral lysis. Antivirus immunity should be favored even if it costs the bacterial cells. Within the temperature ranges inaccessible to grazing predators or within the temperature ranges fully accessible to the grazing predators, temperature changes have little effect on the evolution of the antivirus system (Figure 9).

Besides explaining the thermal distribution, our hypothesis suggests that other environmental factors that severely reduce cellular predator abundance should also affect bacterial mortality and CRISPR-Cas distribution. A generalized prediction of our hypothesis is that bacteria living in extreme environments inaccessible to cellular grazers should carry more CRISPR-Cas systems in their genomes. Weissman et al. (2019) recently found a negative interaction between CRISPR-Cas systems and oxygen availability and hypothesize that oxidative-stress-associated DNA repair processes might interfere with the function of CRISPR-Cas systems. Here, we provide an alternate explanation for their observation by extending our hypothesis. All the well-known cellular predators are aerobic organisms. There are no cellular predators or only a few unknown predators in anoxic environments. Similar to growth temperatures at around 45°C, the transition from an oxygenated to an anoxic environment would substantially reduce the grazing-caused bacterial mortality and indirectly increase the requirement of antivirus immunity.

Besides growth temperature, many other ecological and evolutionary factors that might influence the phylogenetic distribution of CRISPR-Cas systems (Palmer and Gilmore, 2010; Weinberger et al., 2012; Iranzo et al., 2013; Vale et al., 2015; Westra et al., 2015; Trasanidou et al., 2019; Rollie et al., 2020; Wimmer and Beisel, 2020) are beyond the scope of this hypothesis. Even for the thermal distribution of CRISPR-Cas systems, we are open to other possible explanations. A bacteriophage infecting the tropical pathogen *Burkholderia pseudomallei* was found to be temperate at lower temperatures (25°C) and tends to go through a lytic cycle at higher temperatures (37°C) (Shan et al., 2014). At least for type I CRISPR-Cas systems, targeting temperate phages has been demonstrated to be a driving force for the loss of adaptive immunity (Rollie et al., 2020). Therefore, bacteria living in low temperatures might have fewer CRISPR-Cas systems because of the temperate-phage-induced bacterial autoimmunity. However, the temperature-associated switching of the life cycle of the bacteriophage of *B. pseudomallei* is just a piece of isolated evidence. At present, we do not know how many phages in nature have similar temperature-associated switching of life cycle as the bacteriophage of *B. pseudomallei*.

We propose that bacterial cells lose the CRISPR-Cas systems when bacterial mortality mostly comes from predator grazing. However, in natural environments, this does not always happen even below 45°C. Bacterial mortality is destined to be more or less contributed by viral lysis because of the widespread of viruses. In addition, prey bacteria are not entirely passive to be grazed, and they could evolve grazing-resistance capacities, like high motility, large size, and biofilm formation (Matz and Kjelleberg, 2005; Jousset, 2012; Lurling, 2021). In a long-term arms race between prey bacteria and grazing protists/bacteria, the prey bacteria might, in some periods, be free of grazing risk and grazing-caused mortality because of newly evolved grazing-resistance strategies. In this case, viral lysis becomes dominant in bacterial mortality, and the bacteria experience an intense selective pressure to have CRISPR-Cas systems. Therefore, our hypothesis is not exclusive to CRISPR-Cas-rich psychrophilic and mesophilic bacteria.

There is no clear pattern in archaeal CRISPR-Cas abundance along the axis of growth temperature (Figure 7). The difference in CRISPR-Cas distribution between bacteria and archaea might come from the physiological, genomic, or ecological differences between the two domains. It is also possible that random noises resulting from the small sample size have masked the thermal distribution pattern. In a recent analysis on the relationship between growth temperature and GC content using the Topt dataset from the database TEMPURA (Sato et al., 2020), we found that Topt is significantly correlated with bacterial genome GC content (*N* = 681) but not archaeal genome GC content (*N* = 155). Then, we randomly drew 155 bacteria from the 681 bacteria 1000 times. In > 95% rounds of resampling, the positive correlations became statistically non-significant (*p* > 0.05) (Hu et al., 2022). The results suggest that the effective sample size in phylogenetically related data is much smaller than the census number of the analyzed lineages. We hope to replicate the present analysis in archaeal genomes in the future when several hundred or thousands of archaeal species are available.

Our hypothesis states that antivirus immunity is restricted at low temperatures because bacterial mortality mostly comes from grazing. However, sharp increases of antivirus immunity at around 45°C were observed in the CRISPR-Cas systems but not in the RM systems. Here we make further speculation. The intense grazing pressures at low temperatures would favor the acquirement of grazing resistance. Some grazing-resistant genes, like that in type III secretion systems (Matz et al., 2011) and that involved in biofilm formation (Wang et al., 2017), have been found in genomic islands, a widespread tool for horizontal gene transfer (Juhas et al., 2009). In the evolution of grazing resistance, horizontal transfer of the grazing-resistant genes would be selected. Both the CRISPR-Cas systems and the RM systems could depress horizontal gene transfers. However, the RM systems would not stop the transfer among closely related bacteria, especially when they have shared RM systems (Dimitriu et al., 2019). Besides this, the CRISPR-Cas and RM systems differ in many aspects (Dimitriu et al., 2020). We are open to other possible explanations.

## Conclusion

The CRISPR-Cas systems are known to be enriched in thermophilic and hyperthermophilic prokaryotes. In this paper, we take a step further by revealing an abrupt increase of bacterial CRISPR-Cas abundance at around 45°C and putting forward a new hypothesis on the thermal distribution of bacterial CRISPR-Cas systems. Grazing of cellular predators and viral lysis are the primary sources of bacterial mortality; their negative interaction largely influences the tradeoffs between the costs and benefits of antivirus strategies and grazing resistance strategies. As cellular predator diversities and grazing risk precipitously decline at around 45°C, viral lysis becomes the dominant source of bacteria mortality, and the requirement of adaptive immunity might be increased indirectly.

## Data Availability Statement

The datasets presented in this study can be found in online repositories. The names of the repository/repositories and accession number(s) can be found in the article/Supplementary Material.

## Author Contributions

D-KN conceived the study and wrote the manuscript. X-RL and Z-LL performed the analysis. All co-authors have reviewed and approved the manuscript prior to submission.

## Conflict of Interest

The authors declare that the research was conducted in the absence of any commercial or financial relationships that could be construed as a potential conflict of interest.

## Publisher’s Note

All claims expressed in this article are solely those of the authors and do not necessarily represent those of their affiliated organizations, or those of the publisher, the editors and the reviewers. Any product that may be evaluated in this article, or claim that may be made by its manufacturer, is not guaranteed or endorsed by the publisher.

## Footnotes

1
https://ftp.ncbi.nlm.nih.gov/genomes/GENOME_REPORTS/
