# Supplementary material for: Precipitous Increase of Bacterial CRISPR-Cas Abundance at Around 45°C
Source: Front Microbiol. 2022 Mar 1;13:773114. doi: 10.3389/fmicb.2022.773114 (PMC8923349; doi:10.3389/fmicb.2022.773114)
Supplement: Supplementary file 2 [file Data_Sheet_2.PDF]

Supplementary Material

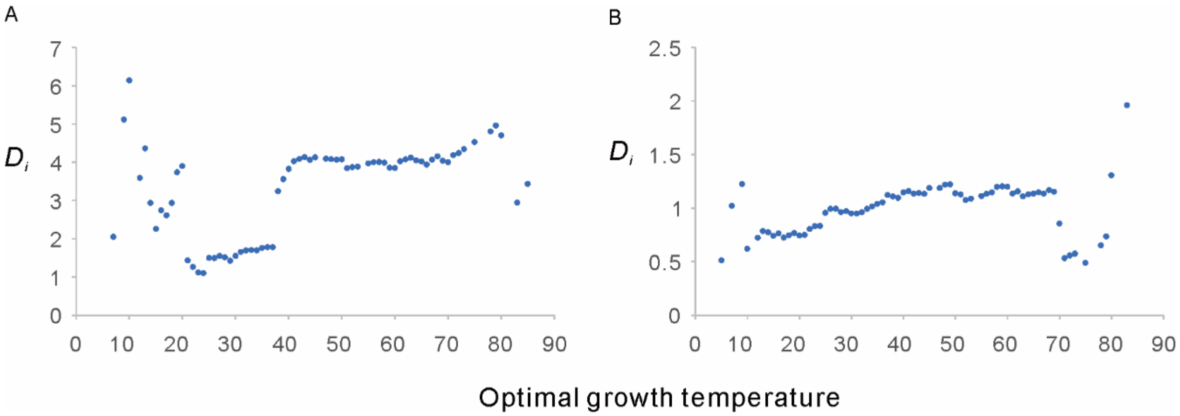

Supplementary Figure 1. Differences (D_i) of bacterial CRISPR array between low and high temperatures. D_i.was defined as the ratio of average CRISPR-Cas abundance at equal and above a temperature divided by that below the temperature. (A) The actual data of the 2944 bacteria. (B) The data was obtained from one of the 1000 rounds of random shuffling.

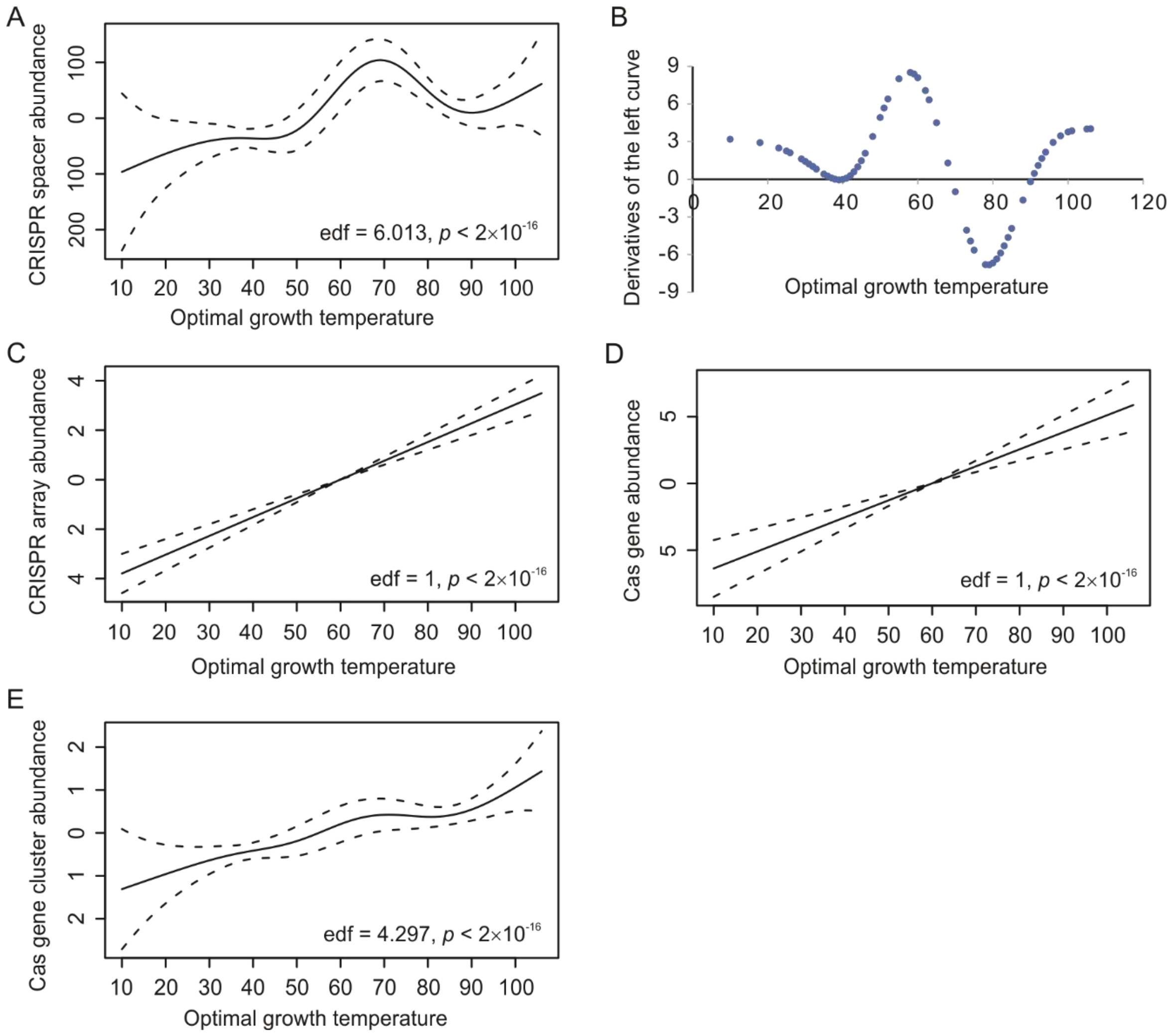

Supplementary Figure 2. The relationship between archaeal optimal growth temperature (Topt) and the CRISPR-Cas abundance revealed by the generalized additive model (GAM). High nonlinearity was detected in the relationship of Topt with CRISPR spacers (A), but not CRISPR arrays (C), *cas* genes (D), and *cas* gene clusters (E). The derivatives of the GAM model curves in (A) revealed the most abrupt transitions at 58°C (B). The effective degrees of freedom (edf) proxy for nonlinearity in the relationships.

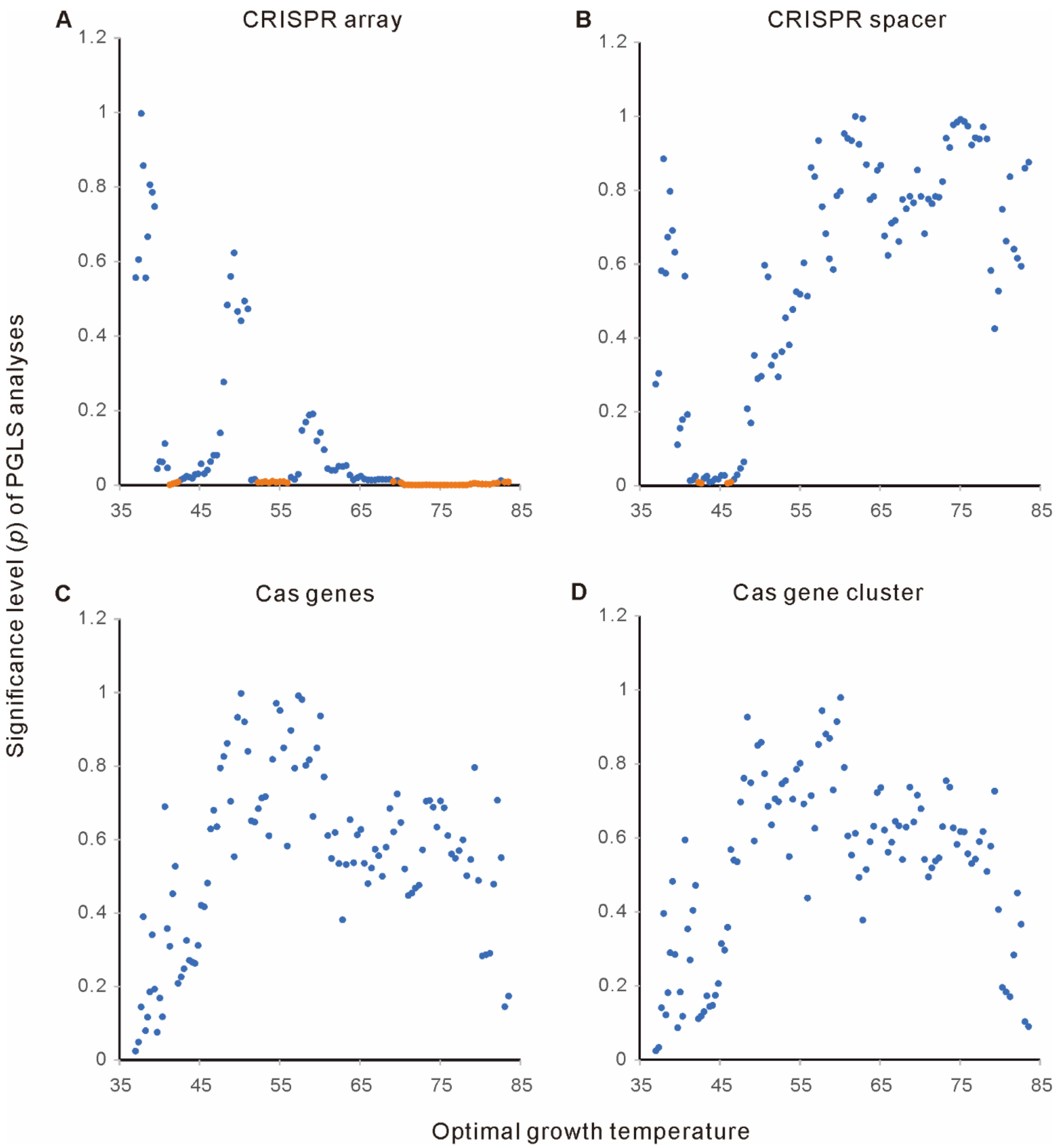

Supplementary Figure 3. Segmental phylogenetic generalized least squares (PGLS) regression analysis of the relationships between CRISPR-Cas abundances and optimal growth temperatures (Topt). The 183 archaea were aligned along the Topt axis. One hundred neighboring samples were taken in each round of PGLS analysis. The average Topt of each 100 samples was presented in the scatter charts. The regression results with positive slopes and < 0.01 significance values are orange.
